# Calcium and calcium-related proteins in endometrial cancer: opportunities for pharmacological intervention

**DOI:** 10.7150/ijbs.68591

**Published:** 2022-01-01

**Authors:** Ting Huang, Jingyi Zhou, Jianliu Wang

**Affiliations:** Department of Obstetrics and Gynecology, Peking University People's Hospital, Beijing 100044, P.R. China.

**Keywords:** Endometrial cancer, Calcium, Estrogen, Calcium channel blocker

## Abstract

Intracellular calcium ions are key second messengers and play an important role in malignant transformation and cancer progression. Estrogen can evoke intracellular calcium increases through membrane-initiated effects and activate subsequent kinase cascades within minutes in normal and cancerous epithelial cells. Ca^2+^-related proteins are expressed in normal epithelial cells or endometrial cancer cells, some of which are upregulated by estrogen. Both estrogen-induced transient calcium increases and long-term changes in protein expression levels may be involved in regulating cancer initiation, progression and metastasis. Calcium channel blockers are reported to regulate both the rapid estrogen-induced intracellular Ca^2+^ increase and cell proliferation, apoptosis and migration, thus having the potential for pharmacological modulators to be repurposed for the treatment of endometrial cancer.

## Introduction

Endometrial cancer (EC) is one of the most common gynecological malignancies worldwide and is a global threat to women's health and well-being. Approximately 420000 patients were newly diagnosed with EC in 2020, accounting for 4.5% of all cases in women 1. Calcium ions are ubiquitous intracellular messengers that regulate a myriad of cellular processes and can affect cell survival. Our previous studies have identified serum calcium ion level as a potential predictor for lymph node metastasis and positive peritoneal cytology in endometrial cancer, suggesting that calcium ions participate in the progression of EC 2, 3.

Calcium homeostasis is a crucial determinant of cellular function and survival. Calcium ions in the cytosol are dynamically regulated by the plasma membrane, endoplasmic reticulum and mitochondria. A sustained calcium increase from extracellular calcium entry or release from calcium stores can affect cancer cell growth, migration, distant metastasis, and survival 4-6. The cytosolic calcium concentration is tightly regulated by ion channels, pumps and exchangers 7. Our previous work suggested that inhibitors of L-type voltage-gated calcium ion channels and transient receptor potential vanilloid 4 (TRPV4) could partly block calcium influx in EC cells 8, 9. In this review, we summarize the latest studies on calcium signaling, differentially expressed calcium-related proteins and their functions in cancer progression as well as the potential for pharmacological application.

### Relationship between estrogen and calcium signal

Clinically, prolonged estrogen stimulation without progesterone antagonism increases the risk of endometrial cancer and estrogen-dependent cases represent 75-90% of all endometrial cancers 10. Our previous work suggested that endometrioid carcinoma had higher serum calcium levels than non-endometrioid carcinoma, the subtype that usually suffers from estrogen deprivation 11. Estrogen is an important hormone that rapidly induces calcium mobilization and regulates calcium-related protein expression in the endometrium 12-14. On the one hand, estrogen stimulus drives calcium influx, and rapidly augmented calcium triggers a series of reactions, which are a main part of non-genomic effects. Any dysfunction of this process may contribute to cancerous transformation and progression. Often, the rapid Ca^2+^ surge causes photophosphorylation of kinase cascades and regulates transcription factors, thus affecting cellular processes and function. On the other hand, long-term estrogen stimulus causes some calcium-related protein changes in normal endometrial epithelial cells (EECs) and cancer cells 14, 15. Sustained high Ca^2+^ exerts its influence on both epithelial cells and the microenvironment 16, 17. However, it remains uncertain whether estrogen-induced short-term effects and long-term regulation of calcium function together in the occurrence and progression of endometrial cancer.

### Estrogen-induced rapid intracellular calcium increases in endometrial cells

**Figure 1:**
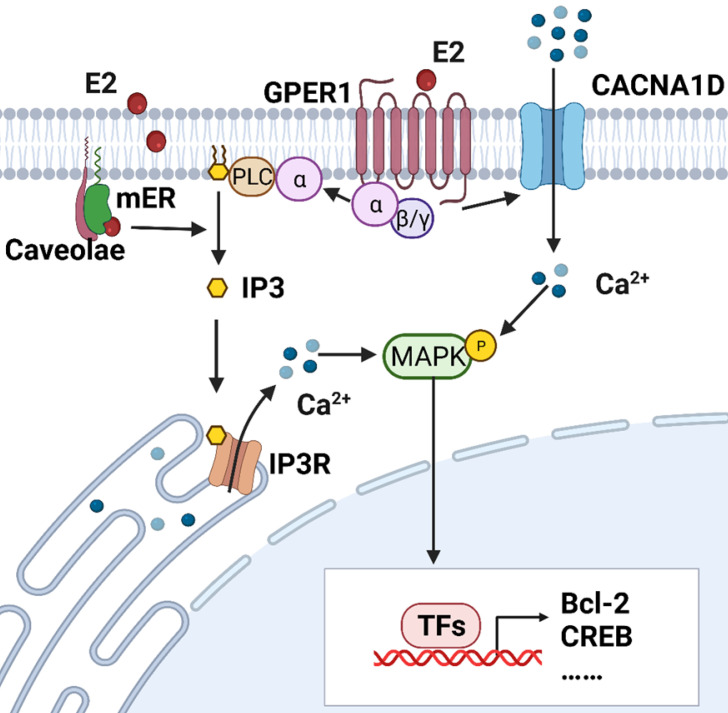
** Estrogen-induced rapid calcium changes in endometrial cells.** Estrogen can rapidly trigger an increase in intracellular calcium in endometrial cells through membrane-initiated signaling. Intracellular calcium mobilization is mediated by different signals: ① GPER1- Gα-CACNA1D; ② GPER1- Gα-PLC-IP3-IP3R-Ca^2+^ store; and ③ mER-PLC-IP3-IP3R-Ca^2+^ store. The degree of increase in cytosolic free Ca^2+^ and duration of maintenance vary in different cells. Increased intracellular calcium activates the MAPK pathway and regulates the expressions of Bcl-2, CREB, and so on. (Created with BioRender.com)

Intracellular calcium is measured by the accumulation of ^45^Ca^2+^ and calcium fluorescent probes, such as fluo-2, fluo-3, fluo-4 and fluo-8. Estrogen-induced rapid increases in intracellular calcium have been detected in endometrial epithelial cells isolated from the uteri of rats and RL95-2 cells (a poorly polarized human endometrial cancer cell line) 12, 13. Moreover, Zhang LL et al demonstrated that both E2 and its membrane-impermeable conjugate, estrogen and bovine serum albumin (E2-BSA), can elicit calcium influx in Ishikawa cells, a moderately polarized endometrial cancer cell line 18. It appears that estrogens may act directly at the cell membrane and exert effects on the activity of ligand-gated ion channels. Transient increases in intracellular free calcium are reported to precede or trigger the cell cycle and growth, which is exactly the end-point of estrogen effects on the endometrium 19. Figure 1 illustrates estrogen-induced intracellular calcium increases in endometrial cancer.

The addition of 17β-estradiol at a final concentration of 1 nM increased ^45^Ca^2+^ uptake, reaching a peak at about 230% of baseline within 30 minutes. ^45^Ca^2+^ uptake was gradually restored to baseline in rat EECs 13. About 15% of RL95-2 cells were responsive to 17β-estradiol stimulus and showed transient intracellular calcium rises within 10 minutes with a maximal value about 130% of baselines obtained at a concentration of 10 nM 12. Intracellular calcium peaked at about 300 seconds and lasted for 600 seconds after adding E2 or E2-BSA to Ishikawa cells. Interestingly, calcium waves showed two peaks under E2-BSA stimulation 18. The different amplitudes and rates may be partly due to the concentration of estrogen.

### Source of estrogen-induced intracellular calcium increase

The estrogen-evoked Ca^2+^ increase may occur as a result of Ca^2+^ entry from the extracellular milieus or Ca^2+^ release from intracellular sources. Morley demonstrated that the estrogen-triggered calcium surge was not affected by incubating the cells in Ca^2+^-free solution or pretreating cells with calcium channel blockers (CCBs) but was abolished by incubating cells with inhibitors of inositol phospholipid hydrolysis in chicken granulosa cells 20. Teresa et al. demonstrated that E2 could induce a rise in intracellular calcium in the presence or absence of extracellular calcium 21. These results imply that estrogen induces Ca^2+^ mobilization mainly from intracellular stores.

However, some studies identified that an estrogen-induced calcium rise had a critical dependence on external calcium. Estrogen was also reported to increase the activity of plasma membrane calcium pumps in distal tubule kidney cells 22. Additionally, E2-induced rapid Ca^2+^ influx in hippocampal neurons and endometrial cancer cells could be significantly inhibited by nifedipine, a calcium channel blocker 18, 23. Wu TW et al found that the initial influx of Ca^2+^ through the L-type calcium channel is necessary for E2 activation of downstream signals 23. Moreover, 17β-estradiol increased intracellular Ca^2+^ in a biphasic manner through extracellular calcium entry and endoplasmic reticulum release in rat osteoblasts 24.

In normal EECs, E2 induced ^45^Ca^2+^ uptake from the medium 13. In RL95-2 cells, 17β-estradiol could not induce calcium transients when the RL95-2 cells were bathed in external Ca^2+^-free medium, implying a dependence on calcium entry. However, the calcium surge was significantly increased by depletion of intracellular calcium stores and decreased after treatment with an inhibitor of protein kinase C (PKC), which suggested that calcium release from intracellular stores via the PKC-sensitive pathway contributed to E2-induced intracellular calcium increases 12. Similarly, E2-BSA elicited a dual peak in Ishikawa cells, the first coming from external calcium influx and the second releasing from intracellular calcium stores 18. In summary, the two sources may also coexist in endometrial cells.

### Receptors that mediate estrogen-induced calcium increase in endometrial cells

There is no consensus regarding which receptors should be responsible for the rapid Ca^2+^ rise. As a membrane-initiated effect, membrane estrogen receptor (mER) is widely investigated. Endometrial cells show abundant binding to estrogen on the cell surface 25. Several variants of ERα and ERβ as well as estrogen receptor G protein-coupled estrogen receptor (GPER1, also known as GRP30) have been reported to be associated with non-genomic estrogen signaling 26-28.

Classical mERs are identical to nuclear estrogen receptor (nER) in not only spectra, weight and affinity to estrogen, but also in their protein epitopes 29, 30. ERα and ERβ were detected in endothelial cell caveolae 31, 32. Without the transmembrane domain, classical ERs may interact with the plasma membrane by covalent binding to membrane proteins such as caveolin-1 33. ERαs translocation to the membrane is dependent on direct binding to caveolin-1. The complex formed by ERα, Src homology and collagen homology (Shc), and insulin-like growth factor receptor-1 (IGF-1R) increases the mERα levels within caveolae rafts of the plasma membrane once stimulated by estrogen 34. In Ishikawa cells, rapid estrogen-induced calcium mobilization could be partly inhibited by the ER antagonist, ICI182780 18. E2/ERα activates phospholipase C (PLC)-dependent inositol 1,4,5-trisphosphate (IP3) production mediated by Gαi/o proteins, thus causing Ca^2+^ store release. E2-induced calcium mobilization was completely blocked by U73122, a PLC inhibitor in ERα-overexpressing COS7 cells 35, 36. In addition, the pattern of testosterone action offers another possibility of regulating calcium flux in prostate cells. The N-terminal region of androgen receptor (AR) has specific sites for transient receptor potential melastatin 8 (TRPM8) and the accumulation of the TRPM8-AR complex in lipid rafts mediates testosterone-induced cell migration 37. A previous study identified several calcium binding sites in the ERα ligand binding domain 38. Moreover, calmodulin regulates the calcium-dependent activation of ERα by directly binding to ERα at several sites 39.

As a novel estrogen receptor, GPER1 is reported to participate in estrogen-triggered non-genomic effects in ovarian cancer 40, ER-negative breast cancer 41 and thyroid cancer cells 42. GPER1 can regulate intracellular free calcium by (1) activating membrane ion channels, (2) regulating Ca^2+^-calmodulin interactions or (3) triggering Ca^2+^ store release 43, 44. In endometrial cancer cells, the GPER agonist G1 facilitated the expression of CACNA1D, while E2-BSA-activated CACNA1D was blocked after silencing the GPER1 gene 8. Likewise, E2 promoted CACNA1D expression in a time-dependent and dose-dependent manner and triggered Ca^2+^ influx through GPER1 in breast cancer cells 45. GPER1 regulates the activity of L-type VGCCs through coupling with Gαs and Gαi/o and triggers subsequent Ca^2+^ entry 43. GPER1 can also be directly regulated by the Ca^2+^-calmodulin complex because of the existence of four distinct calmodulin-binding domains in GPER1, as feedback to the E2-induced calcium increase 46. As a seven-transmembrane receptor, GPER1 stimulates the production of PLCβ and IP3 through coupling with Gβγ. The latter binds to its receptor IP3R1 situated on the endoplasmic reticulum and evokes Ca^2+^ release 47.

Estrogen might directly bind to certain calcium channels. Estrogen, as a lipophilic hormone, affects membrane fluidity, induces membrane fusion and modifies ion channel activity 48. As early as 1986, Kenji et al reported that calcium channels of the estrogen groups in the rat uterus were at a high affinity state, without changing the numbers of calcium channels 49. E2 was reported to directly activate the Maxi-K^+^ channel 50. In hippocampal neurons, estrogen could directly interact with the L-type calcium channel alpha 1C subunit (CACNA1C) at the dihydropyridine site in an estrogen receptor-independent way 51.

### Activated kinase cascades by estrogen-induced calcium signaling

Mitogen-activated protein kinase (MAPK) cascades mainly consist of four pathways: Extracellular-signal-regulated kinase (ERK) 1/2, ERK5, p38 MAPK and c-Jun N-terminal kinase (JNK) 52. E2-induced activation of MAPK cascades can be observed among four endometrial cancer cell lines 12, 18, 53. Accumulating evidences show that E2 can rapidly activate MAPK and in an ER-dependent manner in mammalian cells 54-56. However, the molecular mechanisms underlying E2-triggered MAPK activation and its biological effects remain to be explained.

In MCF-7 breast cancer cells, MAPK activation is preceded by a rapid increase in cytosolic calcium from Ca^2+^ stores in response to estrogen stimulus 21. In Ishikawa cells, either E2 or E2-BSA could induce ERK1/2 phosphorylation 18. Notably, c-Src activation triggered by estrogen could lead to parallel activation of ERK1/2 and Akt signals 57. The same hippocampal neurons were successively stained with a calcium probe and immunocytochemistry for pERK. The results confirmed that the E2-triggered calcium increase was coincident with pERK 23.

Activated MAPKs can translocate into the nucleus and regulate gene transcription, thus playing critical roles in cell proliferation, the cell cycle and apoptosis. Rapid activation of MAPK induced nuclear factor kappa B (NF-κB) activation, CD1 transcription and subsequent cell cycle progression in Swiss 3T3 cells 58. Treatment with a MAPK inhibitor significantly suppressed E2-facilitated proliferation in lactotrophs and breast cancer cells, indicating an important role of the MAPK pathways in E2-dependent progression 30, 59. Ca^2+^/Src/ERK signaling is required for the E2-induced activation of B-cell lymphoma-2 (Bcl-2), an apoptosis regulatory protein 23. Interestingly, E2 results in increased mitochondrial sequestration of Ca^2+^ to attenuate cytosolic Ca^2+^ and a subsequent increase in Bcl-2 expression, aiming to promote mitochondrial tolerance and cell survival in response to glutamate 60. nERs also interacted with MAPK cascades. In MCF-7 cells, ERK2 and ERK5 interacted with different regions of nERα. Upon E2 exposure, activated ERK2 and ERK5 localize with nERα and modulate estrogen-dependent gene transcription and cell proliferation programs 61, 62. In addition to directly binding to ERα, MAPK cascades can indirectly regulate ERα transcriptional activity by targeting several cofactors 63. P38 MAPK and ERK1/2 are involved in hormone-induced activation of c-fos in rat intestinal cells 64. Transient calcium markedly upregulated the expression of semaphorin 3A through the MAPK/activator protein (AP)-1 axis in keratinocytes 65.

There are other E2-induced kinases that are closely associated with the intracellular Ca^2+^ rise. In myometrial cells, the G1-induced intracellular calcium increase occurred prior to myosin light-chain kinase (MLCK) phosphorylation. Thereafter, MLCK became desensitized to Ca^2+^/calmodulin and began dephosphorylation 66. These results described the dynamic change in estrogen-induced calcium and its effects on cell movement. Cytoskeletal rearrangements induced by E2 and tamoxifen could be blocked by a Src inhibitor, implying the important role of Src kinase in estrogen-induced rapid effects in endometrial cancer cells 53.

### Alterations in calcium channels/pumps/exchangers in endometrial cells

**Figure 2:**
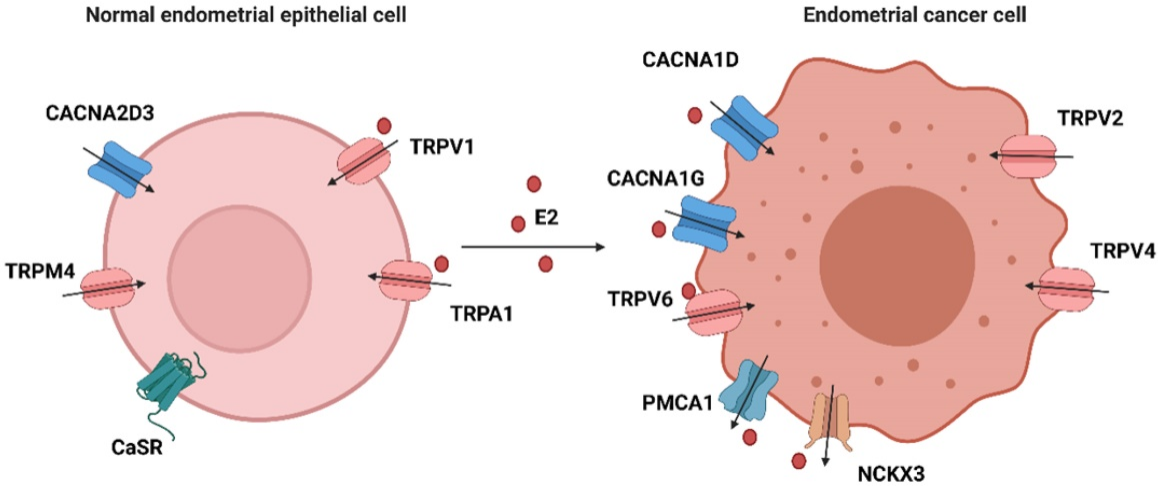
** The expression of calcium-related proteins in EEC and EC cells.** Calcium-related proteins in endometrial cells are divided into four categories: ① that are regulated by E2 in normal epithelial cells: TRPV1 and TRPA1; ② that are regulated by E2 in endometrial cancer cells: CACNA1D, CACNA1G, TRPV6, PMCA1 and NCKX3; ③ that are highly expressed in normal epithelial cells: CACNA2D3, TRPM4 and CaSR; and ④ that are highly expressed in cancer cells: TRPV2 and TRPV4. (Created with BioRender.com)

Cytosolic Ca^2+^ signaling is coordinately controlled by both intracellular and extracellular stores. In most mammalian cells, external stimuli bind to ligand-engaged G protein-coupled receptors (GPCRs), causing subsequent synthesis of IP3 and activation of the IP3 receptor at the endoplasmic reticulum membrane, resulting in the release of calcium from the endoplasmic reticulum 67, 68. Extracellular calcium ions can enter the cytosol through multiple voltage-gated calcium ion channels (VGCC) and transient receptor potential (TRP) family channels. Two main ATP-dependent systems extrude Ca^2+^ from the cytosol: plasma membrane Ca^2+^ ATPases (PMCAs) and sarcoendoplasmic reticular Ca^2+^ ATPases (SERCAs), the former expelling Ca^2+^ to the extracellular space and the latter accumulating Ca^2+^ within the endoplasmic reticulum 69. Together, these calcium channels/pumps/exchangers struggle to maintain dynamic homeostasis. Any dysfunction of these calcium-related proteins may result in a disruption of calcium balance. Therefore, we summarize the expression of calcium-related genes in tissue or cell line(s) of endometrium, their effects on biological behaviors and associations with E2 in Table 1 and Figure 2.

### Voltage-gated calcium channels

Although VCGGs are ubiquitously expressed in excitable cells, they are also detected in many kinds of malignant cells 70. VGCCs are subdivided into L-type, T-type, P/Q-type, R-type and N-type. CACNA1D is an auxiliary member of the alpha-1 subunit family of the VGCC complex and is involved in androgen-stimulated Ca^2+^ influx and androgen receptor transactivation in prostate cancer 71. 17β-estradiol was added to the medium after pretreatment with nifedipine, a blocker targeting CACNA1D and other L-type calcium channels. The mRNA expression of CACNA1D returned to normal at 30 minutes and protein expression started to rise after 60 minutes in Hec-1A cells, suggesting that estrogen regulates the expression of CACNA1D in a rapid manner 72. Next, the effect of CACNA1D on the estrogen-induced intracellular calcium increase was investigated. After knocking down CACNA1D, the intercellular free calcium concentration was significantly reduced in Ishikawa cells compared to the negative control group. Compared to the benign endometrial tissues, atypical hyperplasia and carcinoma tissues have a higher expression of CACNA1D. Moreover, genetic knockdown of CACNA1D inhibited the estrogen-induced growth and migration of Ishikawa cells 8. Calcium channel alpha1G (CACNA1G), a subunit of T-type VGCCs, is also regulated rapidly by estrogen in Hec-1A cells 72.

In contrast, another VGCC, calcium channel alpha 2 delta subunit 3 (CACNA2D3), suppressed cell proliferation and migration, and induced cell apoptosis and Ca^2+^ influx in EC by acting as the downstream of progesterone. The expression of CACNA2D3 was downregulated in EC tissues and cells compared with noncancer tissues or endometrial epithelial cells 73.

### TRP ion channels

TRP ion channels consist of a superfamily of several cation channels (TRPC, TRPV, TRPM, TRPA, TRPP, and TRPML) and can be activated by various stimuli 74, 75. TRP vanilloid 1 (TRPV1) is involved in the reduction in cell viability and the activation of the apoptotic pathway induced by its agonist cannabinoids (CB) in endometrial cancer cells. After exposure to CB, a rapid increase in intercellular calcium levels was detected and a TRPV1 antagonist was able to reverse these effects 76. In rat endometrial cells, about 11% of cells were responsive to capsaicin**,** the TRPV1 activator, and showed a rapid calcium influx. E2 and ERα/ERβ agonists both upregulated TRPV1 mRNA 14. Notably, E2, not E2-BSA, prevented capsaicin from activating TRPV1 channels through ERβ signaling in neurons 77. Therefore, the E2/ERβ complex might regulate TRPV1 activity and modulate rapid calcium entry in some endometrial cells.

TRP vanilloid 2 (TRPV2) has an increased expression in type II endometrial cancer and correlates with worse progression-free survival. Ishikawa cells with TRPV2 overexpression showed a high migratory ability and sensitivity to cisplatin 78.

TRPV4 expression was higher in the EC group than in the normal epithelium group. Furthermore, TRPV4 could regulate migration and metastasis both *in vitro* and *in vivo* through cytoskeleton regulation and the Rho protein pathway 9. In Hec-1A and Hec-1B cells, E2 rapidly induced cytoskeletal remodeling, which was mediated by ERα signaling 53. However, there is no direct evidence indicating that E2 could activate TRPV4 and drive calcium current. In addition, the expression of transient receptor potential vanilloid 6 (TRPV6) was upregulated by E2 in both primary epithelial cells and Ishikawa cells and the increases were completely reversed with an ER antagonist 15.

TRP melastatin 4 (TRPM4) expression has been reported in several cancers and is involved in malignant transformation and immunity modulation 79-81. Bioinformatics analysis based on The Cancer Genome Atlas (TCGA) and Gene Expression Omnibus (GEO) gene expression data of EC tissue and normal endometrial tissue identified TRPM4 as a protective prognostic gene 82. In detail, lower expression of TRPM4 was associated with a higher clinical stage, a more advanced grade, positive lymph node metastasis, myometrial invasion, worse recurrence free survival and overall survival 83. Another membrane of the TRP family, transient receptor potential ankyrin 1 (TRPA1) was also positively regulated by E2 in rat endometrial cells. More studies on its mechanism are needed.

### Ca^2+^ pumps and exchangers

PMCA1 and potassium-dependent sodium/calcium exchanger 3 (NCKX3) are also crucial components of intracellular calcium homeostasis mainly by extruding calcium out of the cytosol. PMCA1 and NCKX3 were richly expressed in the endometrium especially in endometrial and glandular epithelial cells, while their expression was significantly increased at the proliferative phase compared to other phases. PMCA1 and NCKX3 were also detected in Ishikawa cells and their mRNA levels were markedly increased following E2 treatment 15, 84. The E2-induced increase in PMCA1 mRNA levels was completely reversed after pretreatment with ER antagonist 15. All these results indicated that estrogen and its receptors might participate in the regulation of PMCA1 and NCKX3 levels both in endometrial epithelial cells and cancer cells and thus maintain calcium homeostasis.

Other calcium-related genes are involved in the carcinogenesis or progression of EC. Calcium sensing receptor (CaSR) is a membrane of G-protein-coupled receptors. It can activate PLC and respond to intracellular Ca^2+^ fluctuations. CaSR might serve as a tumor suppressor because overexpression induced apoptosis but inhibited invasion of Ishikawa cells 85.

### Ca^2+^ signal and endometrial cancer initiation and progression

Calcium signaling could not only regulate the biological behaviors of cancer cells 86, 87, but also involve the carcinogenic process 88. The oncogenic transformation of epithelial cells is a multistage process during which normal cells shift toward a cancerous state characterized by unlimited proliferation. As we can see in this review, estrogen regulates calcium signaling through rapid calcium influx and alteration of calcium-related protein expression in both normal epithelial and cancerous cells. Therefore, we will discuss the role of Ca^2+^ signals in carcinogenesis and cancer progression (e.g., proliferation, metastasis, cancer cell death and drug resistance).

### Cancer initiation

Both extracellular and intracellular calcium ions have been demonstrated to play important roles in cancerous transformation. The interaction between oncogenic K-Ras and calmodulin is crucial for tumorigenicity through the suppression of the Wnt-Ca^2+^ signaling pathway 89. In addition, intracellular and extracellular calcium ions at high concentrations could enhance ERα transcriptional activity in breast cancer in different ways 90. First, intracellular calcium at physiological concentrations (μM) confers calmodulin (CaM) an active conformation to interact with ERα and enhances receptor-mediated transcription 91. Second, intracellular calcium at hypercalcemic concentrations (mM), may directly bind to ERα 38. Finally, high extracellular Ca^2+^ concentrations (>10 mM) found in metastatic bone lesions increase transcriptional activity of ERα by binding to CaSR at the cell membrane 92. Depleting extracellular calcium in the growth medium by chelation or using calcium-depleted medium inhibited the neoplastic transformation of mouse JB6 epidermal cells. Such a transformation could also be inhibited by nifedipine, an L-type CCB 93. Furthermore, multiple drugs triggering calcium fluxes have been reported to reactivate epigenetically mediated suppression of tumor suppressor genes in colon cancer cells 94. Based on these evidences, the association between CCBs usage (mainly nifedipine, amlodipine, verapamil and diltiazem) and the risk of neoplasia has been widely discussed since the 1990s 95, 96. However, there is no definitive evidence involving the association between cancer and CCBs use.

### Cell proliferation

Calcium ions are closely related to cell proliferation. As early as the 1970s, calcium was thought to be a short regulator of cell growth 97. Interestingly, normal cells require higher external free Ca^2+^ concentrations to induce proliferation than preneoplastic and neoplastic cells 98, 99. External stimuli such as hormones, chemokines and neurotransmitters invoke intracellular calcium increases. Such increases in free calcium ions mainly bind to calmodulin (CaM) and form a Ca^2+^-CaM complex, which subsequently activates calmodulin-dependent protein kinases (CaMK) and leads to the transcriptional activation of NF-κB, nuclear factor of activated T-cells (NFAT) and cAMP response element-binding protein (CREB) 100-102. In addition, CaMs and CaMK were reported to interact with cyclin-dependent protein kinases (CDKs) and regulate cell cycle events, thus affecting cell survival 103, 104.

Calcium influx via CACNA1D in Ishikawa cells was considered to induce the phosphorylation of ERK1/2 and activation of CREB 8. CACNA1G has been proposed as a key regulator of cell cycle progression and survival 105. Mibefradil is a T-type VGCC inhibitor and has been reported to reduce the viability of Hec-1A cells and stimulate proapoptotic factors 72. The downregulation of TRPM4 resulted in an increase in the proportion of AN3CA cells in G2/M phase 83.

### Metastasis

Calcium signaling has been demonstrated to be crucial for regulating processes that occur during metastasis, including cancer cell migration and invasion. Adding calcium to the culture medium increased the migration of Ishikawa and AN3CA cells, which was reversed by a calcium chelating agent 9. Coordinated rearrangements of the cytoskeleton and cell-matrix adhesion are required for cell migration. Sustained or transient increases in intracellular calcium ions activate Ca^2+^-dependent effectors, which can regulate focal adhesion proteins including integrins, paxillin, vinculin, talin, focal adhesion kinase (FAK) and Src family kinases 106. Mechanosensitive TRPV4 channel could interact directly with cytoskeletons and subsequently induce rapid morphological changes 107. Silencing of TRPV4 or pharmacological inhibition with its antagonist modulates the RhoA/ROCK1/LIMK/cofilin pathway and further regulates the actin cytoskeleton and paxillin in Ishikawa cells and ultimately decreases the metastatic ability of this cell line 9.

Degradation of extracellular matrix (ECM) is essential for cancer invasion and distant metastasis. Ca^2+^ influx via TRPV2 can upregulate the expression of some invasive enzymes, such as matrix metalloproteinases and cathepsin B, which can degrade ECM and provide conditions for cancer invasion 108. In addition, Ca^2+^ signaling may regulate epithelial-mesenchymal transition (EMT) induction 109. TRPM4 silencing promotes AN3CA cell progression via the induction of several EMT markers, including E-cadherin, vimentin and N-cadherin 83.

### Cancer cell death

Either intracellular Ca^2+^ overload or perturbation of Ca^2+^ compartmentalization can cause toxicity to the cells and lead to cell death in the form of apoptosis, autophagy and necrosis 110. Therefore, the regulation of activity and expression of certain calcium channels or pumps may be exploited for cancer treatment. It is generally believed that severe calcium dysregulation promotes necrotic death, whereas a moderate Ca^2+^ increase facilitates cell death through autography or apoptosis 111, 112. Massive Ca^2+^ influx results in the activation of hydrolysis enzymes, the subsequent loss of membrane integrity and finally cell death through necrosis 113.

Aberrant Ca^2+^ concentrations always activate endoplasmic reticulum stress (ERS). Stress signals are directly or indirectly relayed from endoplasmic reticulum to the mitochondria and trigger cell apoptosis. On the one hand, Ca^2+^ released form the endoplasmic reticulum activates numerous pathways and subsequently evokes the release of caspase cofactors from mitochondria and promotes cell death 114. On the other hand, the endoplasmic reticulum may communicate with mitochondria by direct contacts at mitochondria-associated endoplasmic reticulum membranes (MAMs) 112. Ca^2+^ handling proteins at the MAM regions control Ca^2+^ transfer and affect cell apoptosis 112.

In addition, Ca^2+^ is a regulator of autophagy. Pharmacological application of the L-type VGCC inhibitor, nifedipine, promotes autophagy through mammalian target of rapamycin (mTOR) and the Beclin1 pathway in Hec-1A cells. Interestingly, adding the autophagy inhibitor 3-MA decreased the protein expression of CACNA1D and augmented nifedipine-induced cell apoptosis, suggesting that autophagy might serve as a protective mechanism for cell survival 115. In summary, CACNA1D inhibitors are considered as potential drug candidates in EC treatment. Cannabidiol increased the expression of cleaved poly ADP-ribose polymerase (c-PARP) and C/EBP homologous protein (CHOP) through TRPV1 activation in Ishikawa cells. c-PARP is an enzyme involved in DNA repair and CHOP plays a key role in ERS-mediated apoptosis.

### Drug resistance

Medications for endometrial cancer mainly include chemotherapeutic drugs and hormone therapy. The platinum-based chemotherapy regimen is the most commonly used in endometrial carcinoma, but the effective rate is not satisfactory 116. Overexpression of TRPV2 increased the cisplatin cytotoxic effect in Ishikawa cells. The TRPV2 activator, cannabidiol, also enhanced the cell-killing effect of cisplatin in TRPV2-transfected cells 78. The detailed mechanism is still unknown. Usually, the mechanisms of chemotherapy resistance involving calcium-dependent pathways include drug efflux, evasion of cell death, increased DNA damage tolerance and dysregulation of certain critical genes 117. Cannabidiol increased the drug retention of several chemotherapeutic agents and synergized with them to induce the apoptosis of glioblastoma cells via TRPV2-dependent Ca^2+^ influx 118. In addition, cannabidiol induced the differentiation of glioma stem-like cells, activated autophagy and overcame carmustine resistance in a TRPV2-dependent manner 119. Cannabidiol was also found to decrease the phosphorylation of nitric oxide synthase 3 (NOS3), increase the production of reactive oxygen species and thus reverse oxaliplatin resistance 120.

Since both rapid and slow regulation of calcium by estrogens exist in endometrial cells, it is interesting to investigate whether calcium ions link these two modes of estrogen. Interestingly, activation of protein kinase A plays an important role in regulating transient receptor potential (TRP) channel functions 121. In addition, a two-pulse regimen of estrogen treatments has been developed to study the association between rapid and slow estrogen actions in human neuroblastoma cells. E2-BSA given in the first pulse for 20 minutes was followed by 17β-estradiol in the second pulse for 2 hours. The results showed that E2-BSA could enhance the transcription of estrogen response element (ERE) initiated by the later administration of 17β-estradiol. This transcriptional activity was blocked by Ca^2+^ chelator, suggesting that calcium plays an important role in coupling the rapid and slow estrogen actions 122. From this perspective, we consider the possibility of inhibiting estrogen actions by using CCBs.

### Modulators of calcium-related proteins

**Figure 3:**
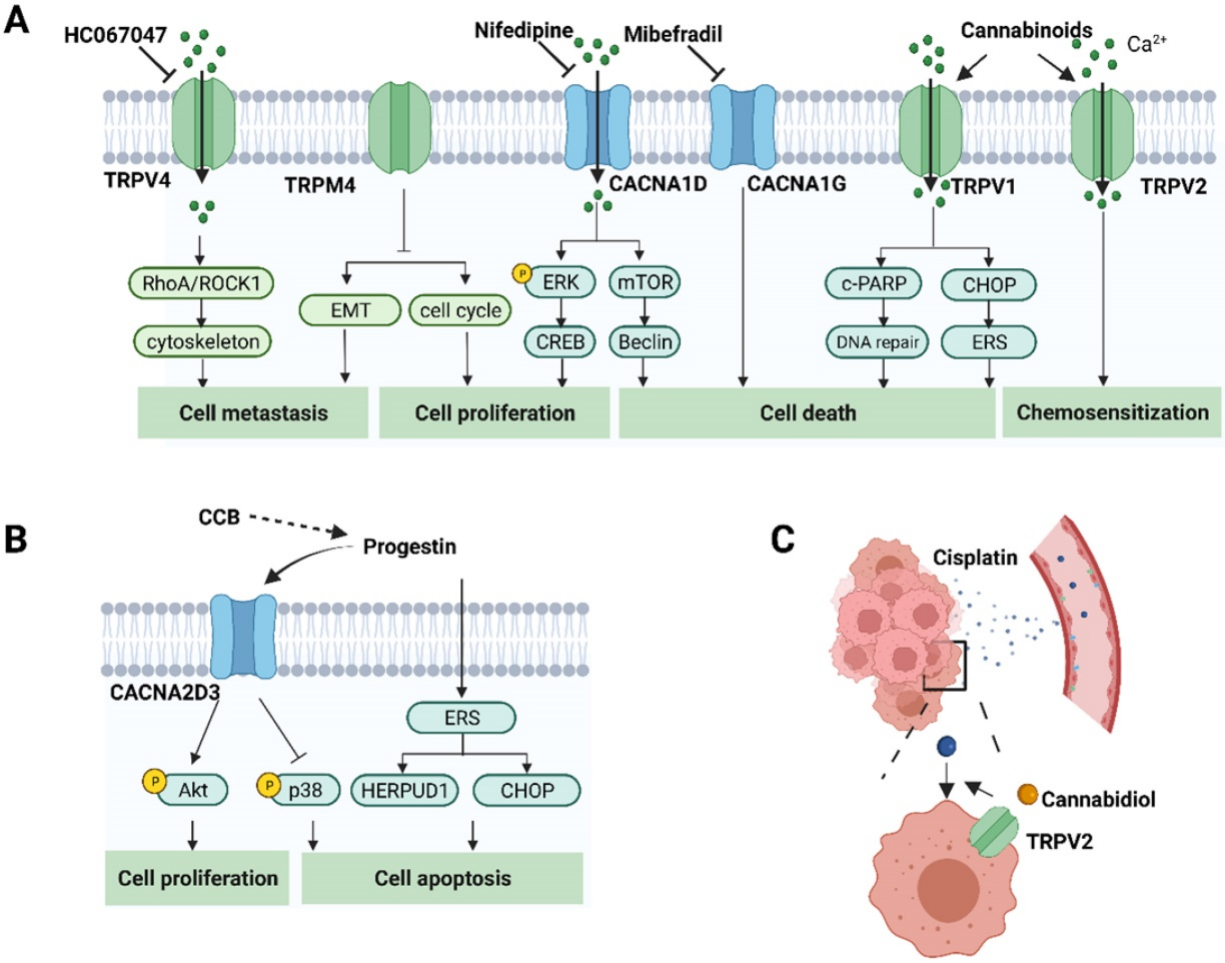
** The role of calcium-related proteins in EC progression and potential strategies for EC treatment. A.** Calcium channels affect cancer cell proliferation, cell death, metastasis and response to chemotherapeutic agents through different pathways in EC. Therefore, inhibitors or activators, have the potential for cancer treatment. It also shows the corresponding modulators of these calcium channels. **B.** Progestin also promotes cell apoptosis by regulating CACNA2D3 and ERS. Calcium channel blockers might generate synergistic anti-tumor effects with progestin in EC. **C.** Calcium channel modulators might enhance the delivery to the tumor site via modulating relaxation of vasculatures. TRPV2 activation by cannabidiol augments the effects of cisplatin in EC cells. (Created with BioRender.com)

Given the involvement of Ca^2+^ signaling in carcinogenesis and progression, specific pharmacological agents modulating Ca^2+^ channels, pumps and exchangers are regarded as druggable. The application of inhibitors or activators depends on whether the resultant alteration to Ca^2+^ promotes cell survival or death 123. Strategies targeting Ca^2+^ signaling in endometrial cancer are illustrated in Figure 3. Pharmacological modulation of calcium channels, pumps or exchangers can affect cell functions and suppress tumor progression by disrupting calcium homeostasis in cancer cells 124, 125. As described above, a variety of calcium permeable ion channels are involved in uterine carcinogenesis and progression. The modulators of calcium channels that are altered in endometrial cancer cells are expected to be potential therapeutic drugs and are outlined in Table 2.

### VGCC inhibitors

Clinically, VGCC inhibitors are used in the treatment of hypertension and other cardiovascular diseases by blocking calcium influx. These drugs are mainly divided into two categories: dihydropyridines (DHPs), such as nifedipine and amlodipine, and non-DHPs, such as diltiazem and verapamil. As increasing evidences suggest the important roles of VGCCs in many cancers, numerous investigators have attempted to repurpose FDA-approved VGCC inhibitors for cancer treatment 126. As early as the 1990s, nifedipine, verapamil and diltiazem were found to inhibit the growth of human astrocytoma U-373 MG cells and human neuroblastoma SK-N-MC cells 127. Mibefradil, a T- and L-type Ca^2+^ channel blocker, was approved by FDA for hypertension in 1997 and then withdrawn due to its interaction with other drugs. Surprisingly, mibefradil showed a promising potential to reduce tumor size and improve the survival rate in glioma animal models and pancreatic cancer xenografts 126, 128. Therefore, mibefradil was repurposed for high-grade glioma cancer and pancreatic cancer treatment. NNC-55-0396, a derivative of mibefradil, was developed to overcome the side effects of its patient and inhibit tumor-induced angiogenesis *in vitro* and *in vivo*, thus appearing to be a promising drug 129.

In our previous studies, nifedipine was involved in the estrogen-induced calcium mobilization and phosphorylation of ERK in Ishikawa cells 18, 130. In addition to the rapid response, nifedipine and mibefradil affect the proliferation, migration and apoptosis of endometrial carcinoma Hec-1A cells 72. Nifedipine also regulated autophagy through the mTOR and Beclin1 pathways in Hec-1A cells 115. These results suggest that these CCBs may serve as drug candidates in targeted therapy of endometrial cancer.

### TRP channel regulators

CBs have been widely studied for their potential anticancer effects since the 1970s 131. In addition to the two G_i/o_-coupled CB receptors, CB1 and CB2, CBs could also pharmacologically target TRPV1, TRPV2, TRPA1 and TRPM8 132. CBs may exert their antitumor effects in a CB1/2-independent manner, as shown in the human pancreatic cancer cell line MIA PaCa-2 133. Several kinds of CBs, including endocannabinoid, anandamide and cannabidiol, have been reported to induce cancer cell death in Ishikawa and Hec50co cells through TRPV1-mediated apoptosis 76. Besides, high TRPV2 expression or its activation by cannabidiol was able to enhance chemotherapeutic drug effects in Ishikawa cells 78.

As TRPV4 was reported to normalize the tumor vasculature *in vivo*, its newly developed agonist, GSK1016790A, may help improve therapy efficacy by augmenting the delivery of cytotoxic agents to the tumor mass 134. Interestingly, TRPV4 exerts an impact on cell migration by regulating the actin cytoskeleton in gastric cancer, ovarian cancer, glioma cancer cells and endometrial cancer 9, 135-137. Pharmacological inhibition with HC067047 or knockdown of TRPV4 inhibits endometrial cancer metastasis, as shown in glioma cells, thus having the potential to be repurposed for EC therapy 9, 137. SOR-C13, a TRPV6 calcium channel inhibitor, significantly reduces ovarian tumor growth *in vivo* and thus enters a phase I human clinical trial in patients with advanced ovarian cancers overexpressing TRPV6 138. HC-030031 is a potent blocker of TRPA1 and can relieve ongoing pain in a breast cancer pain model 139.

### Ca^2+^-ATPase inhibitors

PMCAs play an important role in pumping Ca^2+^ out of the cell, therefore, they can be targeted by certain inhibitors to generate toxic Ca^2+^ concentrations for cell death 140. [Pt(*O*,*O*′-acac)(*γ*-acac)(DMS)], a selective PMCA inhibitor, was shown to alter intracellular calcium homeostasis and trigger rapid apoptosis in MCF-7 cells 141.

### Potential applications of calcium channel modulators for cancer treatment

Currently, the potential application of CCBs is focused on combination with existing treatments, such as chemotherapy or immunotherapy. Targeting Ca^2+^ signaling of stromal cells in the TME, such as immune cells and tumor endothelial cells, is an emerging strategy and can augment the effects of immunotherapy and chemotherapy 134, 142. The synergistic administration of DHPs (lercanidipine and amlodipine) and chemotherapeutic drugs (doxorubicin, vincristine and topotecan) has been reported to induce cell apoptosis and autophagy in gastric cancer cells, neuroblastoma cells and multidrug-resistant leukemia cells 143-145. Verapamil has been well-known to reverse multidrug resistance by directly binding to P-glycoprotein (P-gp) and thus decreasing its expression 146. Another reason for the synergistic efficacy might be that blocking Ca^2+^ signaling in vascular endothelial cells increases the delivery of chemotherapeutic agents to the tumor site. The combination of an activator of TRPV4 with cisplatin could increase the delivery of cytotoxic agents to the tumor site and significantly suppress tumor growth 134. Programmed death 1 (PD-1) and its ligand (PD-L1) are important targets of immunotherapy. Nifedipine and amlodipine could enhance the effects of immunotherapy by depleting PD-L1 expression, and the former even inhibited the expression of PD-1 in T lymphocytes. By mimicking the role of PD-1/PD-L1 inhibitors in tumors, CCBs cooperate with anti-PD-1 therapy in breast cancer, colorectal cancer and colon cancer 142, 147. Chemotherapy and immunotherapy are also important adjuvant therapeutic methods for endometrial cancer. Appropriate antihypertensive drugs in patients with hypertension might benefit cancer treatment.

Progestin is a viable option for fertility-sparing treatment of patients with early EC and palliative treatment of women with advanced EC. Progestin is reported to rapidly activate intracellular calcium increases in multiple cancer cells, such as triple negative breast cancer 148, oral squamous cancer 149 and endometrial cancer 73. Ishikawa cells treated with medroxyprogesterone acetate (MPA) showed increased activation of the ERS pathway. Meanwhile, the ERS-related molecules, CHOP and HERPUD1, were significantly upregulated 150. Alterations in intracellular calcium concentrations often induce ERS and activate downstream pathways. Severe and prolonged ERS leads to cell death. The VGCC inhibitors, verapamil and mibefradil were reported to facilitate cell death via ERS activation in myeloma cells and C2C12 myoblasts 151, 152. Furthermore, progesterone inhibited cell growth and promoted apoptosis via CACNA2D3 in Ishikawa cells 73. In addition, four progesterone derivatives were reported to have binding sites on P-gp, which were distinct and nonexclusive with the modulating sites of verapamil. Progesterone, in combination with verapamil, exhibited synergetic activities to induce P-gp ATPase activity and further reverse MDR in a highly resistant tumor cell line 153. These results provide a foundation for future application of coadministration of progesterone and calcium channel blockers.

The application of CCBs in endometrial cancer treatment depends on a variety of factors. The tissue distribution of calcium channels/pumps/exchangers and possible side reactions might be an important consideration. Drugs targeting certain factors with widespread expression are likely to be associated with generalized toxicity, as they will damage normal cells. After all, VGCC inhibitors have a long history in the clinical treatment of hypertension by blocking VGCCs on vascular endothelial cells. In the majority of studies, repurposing VGCC inhibitors for cancer treatment alone is usually at a much higher dose than is traditionally used to treat hypertension. Therefore, new drug delivery and formulation methods should be exploited. Nanoscale therapeutic delivery systems wrapping CCBs are expected as potential future medicines by increasing accumulation at the tumor site.

## Conclusion

Dysregulated Ca^2+^ homeostasis plays an important role in the occurrence and progression of endometrial cancer. E2 rapidly induces an increase in intracellular calcium and upregulates some of the calcium channels/pumps/exchangers afterwards. Membrane estrogen receptors and downstream kinase cascades participate in the rapid response and affect cell function mainly by activating gene transcription. The role of the Ca^2+^ signaling in tumor onset and progression goes beyond the cancer cell itself and may also involve the regulation of the TME. Certain calcium channel modulators are involved in both rapid E2-induced intracellular calcium increases and Ca^2+^-related biological behavior changes in endometrial cancer. It has the potential for CCBs to be repurposed alone or in combination with existing toxic agents for endometrial cancer therapy. Furthermore, structure-based rational transformation of CCBs, aiming to target specific cancer cells and reduce their side effects, will likely provide promising leads for EC treatment in the future.

## Figures and Tables

**Table 1:** Altered Ca^2+^ channels/pumps and their functions in endometrial cells

	Calcium channel/pumps	Differential expression in EC tissues comparing to normal	Effects on biological behavior	E2 effects on Ca^2+^ channels/pumps		ref
				E2 stimulation	Cell line(s)	
VGCC	CACNA1D	↑	promotes proliferation, migration and apoptosis and estrogen-induced Ca^2+^ influx in ISK cells	↑	ISK	8
	CACNA1G	ND	promotes proliferation, migration and apoptosis	↑	ISK	72
	CACNA2D3	↓	suppresses cell proliferation and migration, and induce apoptosis and Ca^2+^ influx in ISK and RL95-2 cells	ND	ND	73
TRP	TRPV1	ND	reduces viability of Ishikawa and Hec50co cells	↑	Rat EEC	14
	TRPV2	↑ in non-endometrioid tissues	promotes migration and chemo-sensitivity in ISK cells	ND	ND	5
	TRPV4	↑	promotes migration in ISK and Hec-1A cells	ND	ND	9
	TRPV6	ND	ND	↑	ISK	15
	TRPM4	↓	suppresses proliferation and migration in AN3CA cells	ND	ND	83
	TRPA1	ND	ND	↑	Rat EEC	14
Pump(s)	PMCA1	ND	ND	↑	ISK	15
	NCKX3	ND	ND	↑	ISK	84

↑ increased levels in cancer sample; ↓ decreased levels in cancer samples; ND, not determined; ISK, Ishikawa; EEC, endometrial epithelial cell; EC: endometrial cancer; VGCC: Voltage-gated calcium ion channels; CACNA1C: Calcium channel alpha1C; CACNA1D: Calcium channel alpha1D; CACNA1G: Calcium channel alpha1G; CACNA2D3: Calcium channel alpha 2 delta subunit 3; TRP: Transient receptor potential; TRPV1: TRP vanilloid 1; TRPV2: TRP vanilloid 2; TRPV4: TRP vanilloid 4; TRPV6: TRP vanilloid 6; TRPM4: TRP melastatin 4; TRPA1: TRP ankyrin 1; PMCAs: Plasma membrane Ca^2+^ ATPases; NCKX3: potassium-dependent sodium/calcium exchanger 3.

**Table 2:** Studies on drugs targeting EC-related Ca^2+^ channels/ exchangers/ pumps in cancer cells

Target	Drug	Activator/Inhibitor	Effects on EC		Effects on other cancer cells	
			Studies in EC cells	ref	Studies in non-EC cells	ref
L-type VGCC	Nifedipine	inhibitor	Nifedipine reduced the proliferation, invasion, apoptosis and promoted autophagy in Hec-1A cells	72,115	Nifedipine suppressed colon cancer progression	147
T-type VGCC	Mibefradil	inhibitor	Mibefradil reduced the proliferation, invasion, apoptosis in Hec-1A cells	72	Inhibited proliferation and induced apoptosis in leukemia cells and glioblastoma cells.	126,128
	NNC 55-0396	inhibitor	ND		NNC 55-0396 suppressed tumor growth in glioblastoma	129
TRPV1/2	Cannabinoids	inhibitor	Cannabinoids reduced cell viability, activated apoptosis in type I cells (Ishikawa, MFE-280, HEC-1a and PCEM002 cell lines) and autophagy in mixed type EC cells (PCEM004a and PCEM004b cell lines), inhibited migration ability of ISK, PCEM004a and PCEM004b cells and improved chemotherapeutic drugs cytotoxic effects in ISK cells.	76, 78	Cannabinoids inhibited cell growth, migration and invasion of several cancer types, including brain, breast and prostate cancers	132-133
TRPV4	GSK1016790A	activator	GSK1016790A increased motility of ISK cells	9	GSK1016790A reduced the proliferation of tumor endothelial cells	134
	HC067047	inhibitor	HC067047 led to decreased motility of Hec-1A cells and peritoneal spreading sites *in vivo*.	9	HC067047 suppressed glioma migration and invasion	137
	SOR-C13	inhibitor	ND		SOR-C13 reduced ovarian tumor growth in a mouse model.	138
TRPA1	HC-030031	inhibitor	ND		HC-030031 alleviated pain in cancer patients.	139
PMCA	[Pt(*O*,*O*′-acac)(*γ*-acac)(DMS)]	inhibitor	ND		[Pt(O,O′-acac)(γ-acac)(DMS)] triggers rapid apoptosis in MCF-7 cells.	141

ND, not determined; EC: endometrial cancer; VGCC: Voltage-gated calcium ion channels; TRPV1: TRP vanilloid 1; TRPV2: TRP vanilloid 2; TRPV4: TRP vanilloid 4; TRPA1: TRP ankyrin 1; PMCAs: Plasma membrane Ca^2+^ ATPases.

## Abbreviations

EC: Endometrial cancer
TRPV4: Transient receptor potential vanilloid 4
EEC: Endometrial epithelial cells
E2-BSA: Estrogen and bovine serum albumin
CCBs: Calcium channel blockers
PKC: Protein kinase C
mER: Membrane estrogen receptor
nER: nuclear estrogen receptor
GPER1: G protein-coupled estrogen receptor
Shc: Src homology and collagen homology
IGF-1R: Insulin-like growth factor receptor-1
PLC: Phospholipase C
IP3: Inositol 1,4,5-trisphosphate
MAPK: Mitogen-activated protein kinase
ERK: Extracellular-signal-regulated kinase
AP: Activator protein
Bcl-2: B-cell lymphoma-2
JNK: c-Jun N-terminal kinase
NF-κB: Nuclear factor kappa B
MLCK: Myosin light-chain kinase
CaM: Calmodulin
CaMK: Calmodulin-dependent protein kinases
NFAT: nuclear factor of activated T-cells
CREB: cAMP response element-binding protein
CDKs: cyclin-dependent protein kinases
FAK: focal adhesion kinase
ECM: Extracellular matrix
EMT: Epithelial-mesenchymal transition
ERS: Endoplasmic reticulum stress
MAM: mitochondria-associated endoplasmic reticulum membranes
mTOR: Mammalian target of rapamycin
c-PARP: poly ADP-ribose polymerase
CHOP: C/EBP homologous protein
NOS3: Nitric oxide synthase 3
TME: Tumor microenvironment
CAFs: Cancer-associated fibroblast cells
GPCR: G protein-coupled receptors
AR: Androgen receptor
VGCC: Voltage-gated calcium ion channels
CACNA1C: Calcium channel alpha1C
CACNA1D: Calcium channel alpha1D
CACNA1G: Calcium channel alpha1G
CACNA2D3: Calcium channel alpha 2 delta subunit 3
TRP: Transient receptor potential
TRPM8: Transient receptor potential melastatin 8
TRPV1: TRP vanilloid 1
TRPV2: TRP vanilloid 2
TRPV4: TRP vanilloid 4
TRPV6: TRP vanilloid 6
TRPM4: TRP melastatin 4
TRPA1: TRP ankyrin 1
ER: Endoplasmic reticulum
PMCAs: Plasma membrane Ca^2+^ ATPases
SERCAs: Sarcoendoplasmic reticular Ca^2+^ ATPases
NCKX3: potassium-dependent sodium/calcium exchanger 3
CaSR: Calcium sensing receptor
CB: Cannabinoid
TCGA: the Cancer Genome Atlas
GEO: Gene Expression Omnibus
DHPs: Dihydropyridines
PD-1: Programmed death 1
PD-L1: Programmed death ligand 1
